# Direct Midline Diastema Closure with Composite Layering Technique: A One-Year Follow-Up

**DOI:** 10.1155/2016/6810984

**Published:** 2016-01-06

**Authors:** Bora Korkut, Funda Yanikoglu, Dilek Tagtekin

**Affiliations:** Department of Restorative Dentistry, Faculty of Dentistry, Marmara University, 34746 Istanbul, Turkey

## Abstract

*Objective*. Maxillary anterior spacing is a common aesthetic complaint of patients. Midline diastema has a multifactorial etiology such as labial frenulum, microdontia, mesiodens, peg-shaped lateral incisors, agenesis, cysts, habits such as finger sucking, tongue thrusting, or lip sucking, dental malformations, genetics, proclinations, dental-skeletal discrepancies, and imperfect coalescence of interdental septum. Appropriate technique and material for effective treatment are based on time, physical, psychological, and economical limitations. Direct composite resins in diastema cases allow dentist and patient complete control of these limitations and formation of natural smile.* Clinical Considerations*. In this case report a maxillary midline diastema was closed with direct composite resin restorations in one appointment without any preparation. One bottle total etch adhesive was used and translucent/opaque composite resin shades were layered on mesial surfaces of the teeth that were isolated with rubber dam and Teflon bands. Finishing and polishing procedures were achieved by using polishing discs. Patient was informed for recalls for every 6 months.* Conclusions*. At one-year recall no sensitivities, discolorations, or fractures were detected on teeth and restorations. Direct composite resins seemed to be highly aesthetic and durable restorations that can satisfy patients as under the conditions of case presented.

## 1. Introduction

Maxillary anterior spacing or diastema is a common aesthetic complaint of patients [1]. Keene described midline diastema as anterior midline spacing greater than 0.5 mm between the proximal surfaces of adjacent teeth [2]. It was reported that maxilla has a higher prevalence of midline diastema than mandible [3]. The midline diastema has a multifactorial etiology. In addition to the labial frenulum, microdontia, mesiodens, peg-shaped lateral incisors, lateral incisor agenesis, cysts in the midline region, habits such as finger sucking, tongue thrusting, and/or lip sucking, dental malformations, genetics, maxillary incisor proclination, dental-skeletal discrepancies, and imperfect coalescence of the interdental septum should be considered factors that can cause diastema [4, 5]. The width to length ratio of the central incisors for aesthetic rehabilitation in complex midline diastema closure cases determines the treatment plan. Decisions such as the amount of distal proximal reduction, the number of teeth to be treated, the placement and location of prominences and concavities to create the illusion, and the decision for full-veneers or just adding to the interproximal are decided according to that ratio [6]. The appropriate technique and material for a patient are also based on time, physical, psychological, and economic limitations [7, 8]. Direct composite resins in diastema closure cases allow dentist and patient complete control in formation of natural smile [9]. Improved materials and techniques are often introduced leading professionals to endless improvement while fulfilling their patients' aesthetic demands [10]. Recent aesthetic composite resin materials have similar physical and mechanical properties to that of the natural tooth and possess an appearance like natural dentin and enamel [11]. They offer an expanded range of shades and varying opacities designed specifically for layering technique whereas early brands of composite resins offered only “body” shades and appeared dull and dense [11–13].

This case report describes direct aesthetic midline diastema closure with composite layering technique.

## 2. Case Report

**Figure 1:**
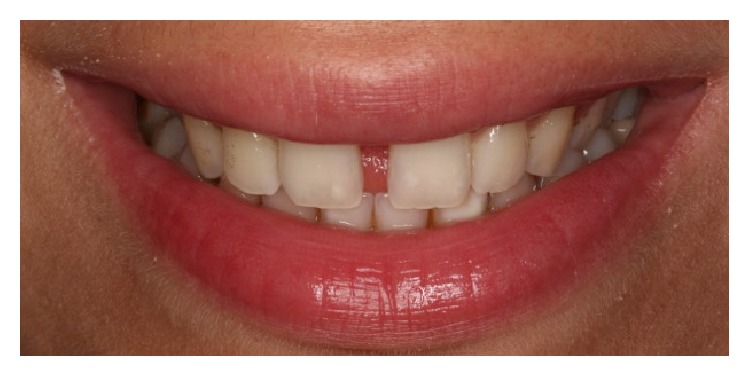
Preoperative extraoral view of the patient with aesthetic problems due to the tongue thrust.

A 32-year-old male patient reported to the Department of Restorative Dentistry, Faculty of Dentistry, Marmara University, with the chief complaint of spacing in the upper front tooth region. Patient's medical history did not reveal any systemic diseases and intraoral examination revealed presence of midline spacing between maxillary central incisors (~4 mm) due to tongue thrust parafunction (Figure 1). No dental caries were observed in both clinical and radiographical examinations. As a more conservative, economical, aesthetic, and quicker option, direct aesthetic partial composite laminate veneers as build-ups for both maxillary central incisors were considered.

**Figure 2:**
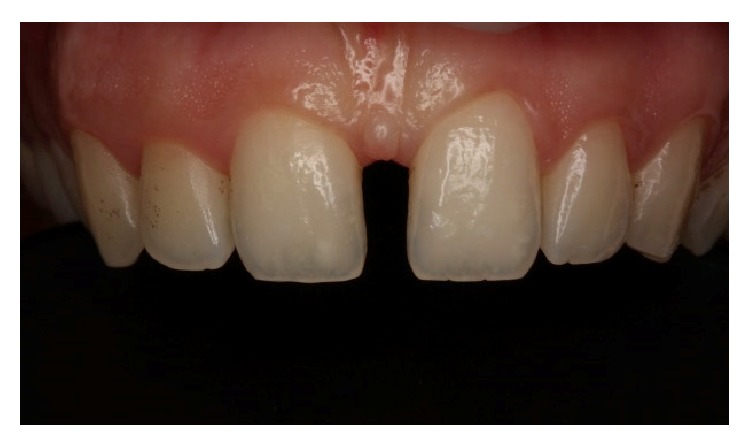
Preoperative intraoral view of the patient and the midline diastema.

**Figure 3:**
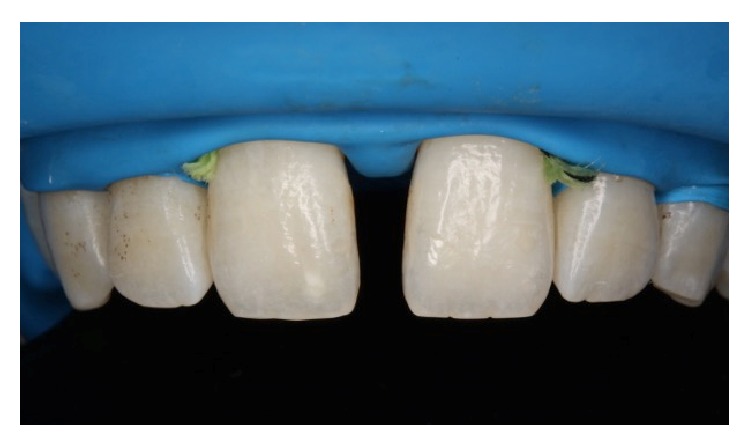
Maxillary anterior teeth were isolated with rubber dam and the central incisors were retracted with retraction cord. No preparations were achieved.

**Figure 4:**
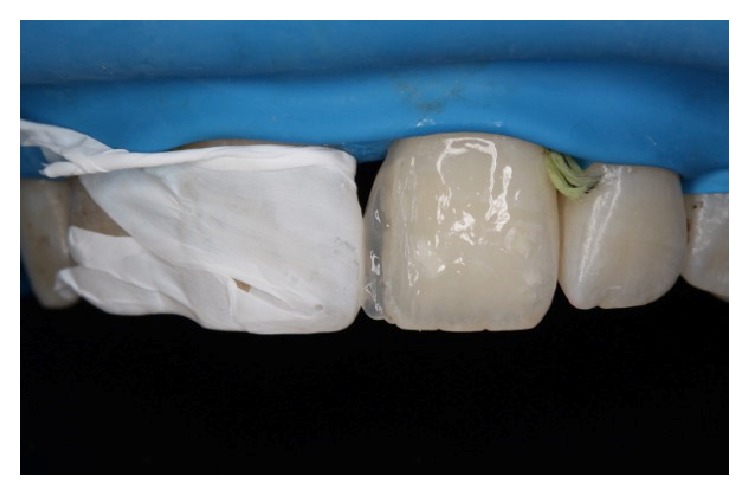
The adjacent central incisor was covered with Teflon band while the other was restored. A thin layer of JE shade transparent composite resin was used palatinally as enamel.

**Figure 5:**
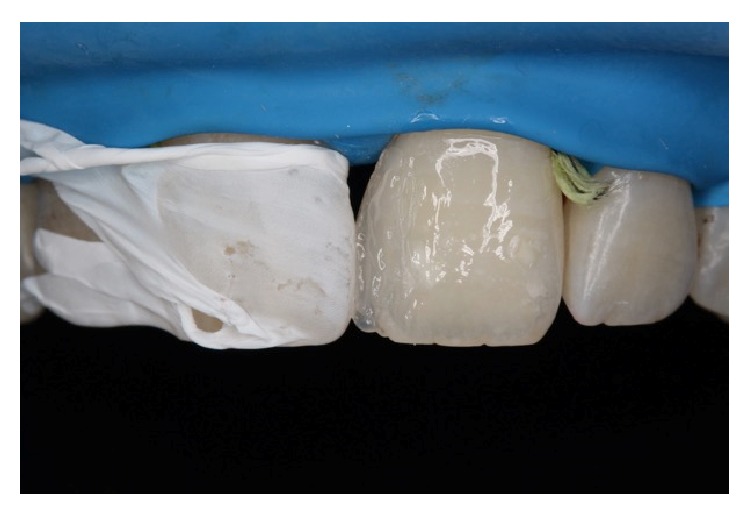
A thin layer of BW shade opaque composite resin was placed roughly as the second layer.

**Figure 6:**
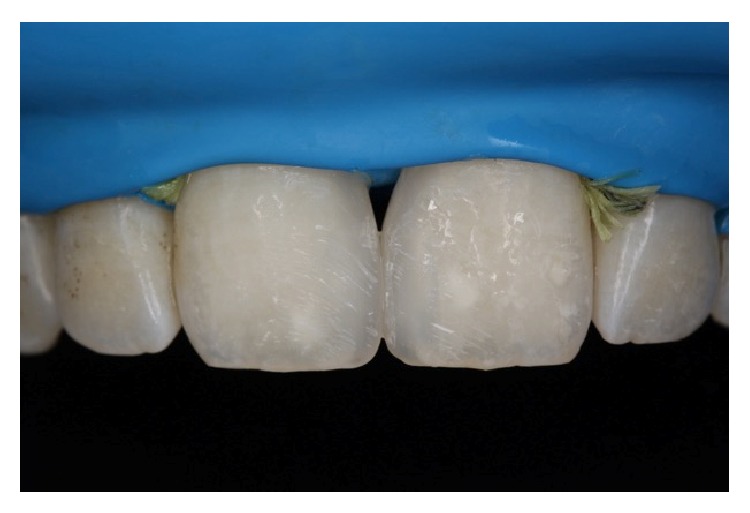
A1 shade composite resin was used as dentin layer and a thin layer of JE shade was used as the top enamel layer. Labial surfaces of the restorations were flattened by using a red banded knife-edge tip diamond bur.

**Figure 7:**
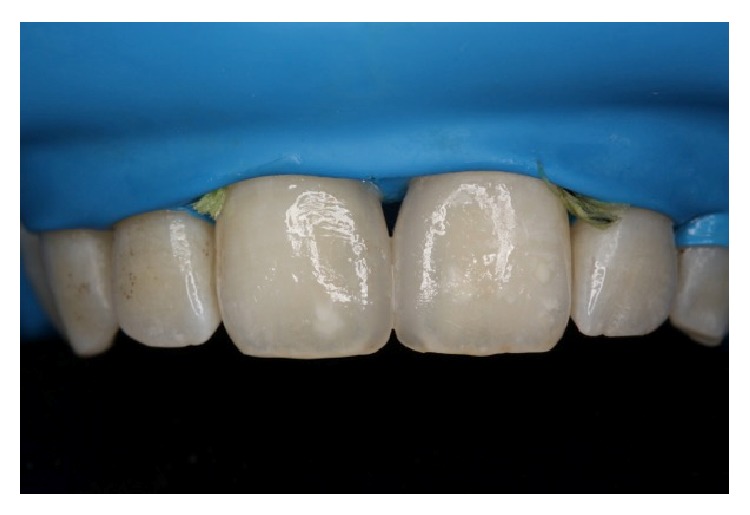
Polishing discs were used for detailed polishing from rough to fine grit.

**Figure 8:**
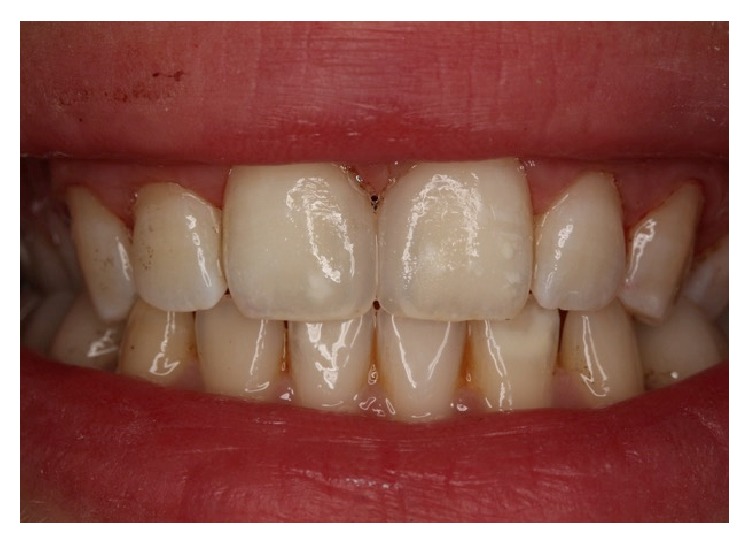
Postoperative view of the restorations just after removal of the rubber dam and the retraction cords.

Firstly, shade selection was considered A1 shade of Vita guide for the teeth to be restored. In order to simulate a natural A1 shade outlook, the shades BW, A1, and JE (Gaenial, GC, Japan) were decided to be used together as layers. No preparations were performed before the restoration procedure (Figure 2). All maxillary incisors were isolated with rubber dam (Kerr, USA) and the central incisors were retracted by using retraction cord (Figure 3). The adjacent central incisor was covered with Teflon band while the other was restored. 37% phosphoric acid (Etching Gel, Kerr, USA) was applied on the mesial surface to be restored for 15 seconds, rinsed for 20 seconds, and dried with air slightly. Then a single bottle bonding agent (Adper Single Bond, 3M ESPE, USA) was applied and polymerized for 20 seconds with a LED light generator (Demi Led Light Curing System, Kerr, USA). A thin layer of JE shade transparent composite resin was used palatinally as enamel (Figure 4). A thin layer of BW shade opaque composite resin was placed roughly as second layer (Figure 5). A1 shade composite resin was used as dentin layer and a thin layer JE shade was used as the top enamel layer. Labial surfaces of the restorations were flattened by using a red banded knife-edge tip diamond bur (Acurata, Germany) (Figure 6). Polishing discs (Ultra Gloss Composite Polishing System, Axis, USA) were used for detailed polishing from rough to fine grains by using a low speed handpiece (DURAtec 2068D, Germany) (Figures 7 and 8).

**Figure 9:**
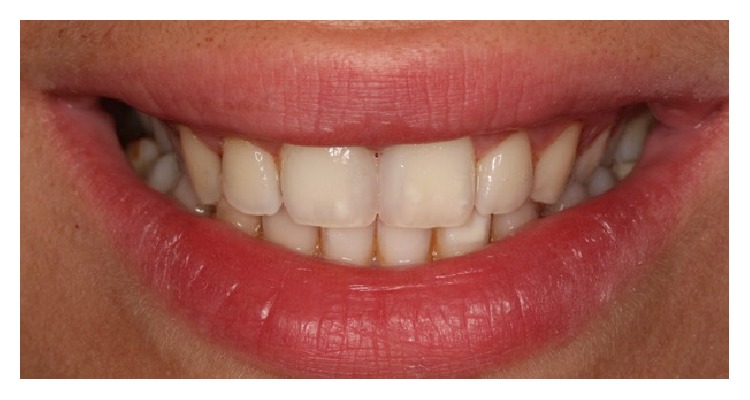
The view of the restorations at one-year recall.

The patient was motivated for oral hygiene and informed for recalls. At the 6-month recall the restorations were just polished with polishing discs. At one-year recall no sensitivities, discolorations, or fractures were detected on the teeth and the restorations (Figure 9).

## 3. Discussion

The direct composite resin restorations can be placed in a single visit, often do not require preliminary models or wax-ups, and do not involve laboratory fees that escalate costs. In terms of aesthetic dentistry, these restorations offer numerous advantages that other possible treatment options such as ceramic veneers and orthodontic treatment do not have. They are kinder to the opposing dentition compared to ceramic materials [14] and, in the event of an unforeseen fracture, they can be repaired easily compared to costly and time-consuming repairs or remakes for porcelain alternatives [15]. There are also some disadvantages of direct composite resin restorations compared to some indirect porcelain alternatives. Most composite materials possess less fractural toughness, shear, and compressive strength and are not ideally suited for ultra-high-stress areas found in certain clinical situations [16, 17]. Presence of unmanaged parafunctional forces such as bruxism, Class III end-to-end occlusal schemes, or noxious oral habits such as nail biting can potentially jeopardize the longevity of direct composite resin restorations [12, 18]. Moreover, the color stability of direct composite resin restorations is not as inert as glazed ceramics; however, this depends on the quality of finishing and polishing procedures and can be prevented with recalls [19, 20]. Regardless of the fact that direct composite resin restorations have these disadvantages, the developing adhesive techniques and better quality resin materials give dentists the chance to create more conservative, functional, aesthetic, economic, and long lasting restorations also in a very short chair time [21, 22].

In this case report, one-year recall of a midline diastema closure treatment by using direct composite resins was assessed. Creating a wax-up restoration previously to simulate the diastema closure and building a silicon matrix to guide final composite resin restoration are a common method for this kind of cases [8, 22]. However, in this case report, another technique was used without creating a silicon matrix. The midline diastema was closed by building up the mesial surfaces of central incisors one by one. The teeth were isolated with rubber dam, retraction cords, and the central incisor adjacent to the one to be restored was covered with Teflon band. Teflon band is a very thin band that gives the opportunity to create very close contact and perfect isolation where resin based restoration materials do not adhere. There was no need for using transparent matrix bands or wedges in this technique. This isolation technique allowed us to create two separate restorations having a really close natural alike contact without creating a dark triangle because of not using wedge which are important advantages compared with the silicon matrix technique [8]. The teeth were restored one by one by layering technique that can also be used in silicon matrix technique [13]. The treatments without creating previous wax-up restorations and a silicon matrix were performed in a very short period of time which is another advantage. In this technique the positioning of the midline and the location of the contact area were decided by the dentist allowing him/her to simulate a natural outlook. However, these decisions and restorations without a guide such as silicon matrix are not easy to perform. The dentist should be well experienced about this technique in order to create a correct midline as well as a natural smile design which can be calculated as a disadvantage compared with the other technique.

Restoration using ceramic fragment is another treatment option for these cases. It is an indirect ceramic restoration that is prepared in a laboratory and attached to previously prepared area of tooth. This technique needs at least two appointments which can be defined as a disadvantage compared with direct techniques. According to the manipulation of the technician, the restorations by using ceramic fragments can be functional and simulate natural esthetics [11]. On the other hand recent studies also showed that direct composite resin restorations are considered functional, stable, aesthetical, and cheaper restorations completed in less chair time by using appropriate techniques for patients with appropriate occlusion [8, 13]. Capability of being repaired easily in case of fractures is another important advantage of direct composite resin restorations. Although direct resin restorations are considered to be stable, the color stability of ceramic restorations is still much better. The best solution for this problem is the perfection in finishing and polishing procedures and frequent recalls [8, 21]. In this case report direct composite resin restorations were decided as the treatment method due to aesthetical demands of the patient having restricted time and money.

At six-month and one-year recalls the general outlook of the maxillary anterior teeth was considered natural and aesthetical. Clinically, both restorations have no fractures and also the restoration margins on both maxillary central incisors demonstrated no discolorations. Although one-year follow-up does not seem long enough and further long term follow-ups are required, restoration problems such as marginal leakage, discolorations, fractures, and debonding for composite resins generally merge within 6 months after the treatment. By taking this into consideration and according to the positive results, an experienced dentist with proper case selection, using an appropriate technique and modern materials, can perform highly aesthetic and durable direct composite resin restorations that can satisfy patients as under the conditions of the case presented.

## References

[1] Koora K., Muthu M. S., Rathna P. V. (2007). Spontaneous closure of midline diastema following frenectomy. *Journal of Indian Society of Pedodontics and Preventive Dentistry*.

[2] Keene H. J. (1963). Distribution of diastemas in the dentition of man. *American Journal of Physical Anthropology*.

[3] Kaimenyi J. T. (1998). Occurrence of midline diastema and frenum attachments amongst school children in Nairobi, Kenya. *Indian Journal of Dental Research*.

[4] Weber (1972). *Quoted in: Orthodontic Principles and Practice*.

[5] Tanaka O. M., Morino A. Y. K., Machuca O. F., Schneider N. Á. (2015). When the midline diastema is not characteristic of the ‘ugly duckling’ stage. *Case Reports in Dentistry*.

[6] Blitz N. (1996). Direct bonding in diastema closure—high drama, immediate resolution. *Oral Health*.

[7] Chalifoux P. R. (1996). Perception esthetics: factors that affect smile design. *Journal of Esthetic and Restorative Dentistry*.

[8] Prabhu R., Bhaskaran S., Prabhu K. G., Eswaran M., Phanikrishna G., Deepthi B. (2015). Clinical evaluation of direct composite restoration done for midline diastema closure-long-term study. *Journal of Pharmacy and Bioallied Sciences*.

[9] Dale B. G., Aschheim K. W. (1993). *Esthetic Dentistry: A Clinical Approach to Techniques and Materials*.

[10] Lee Y.-K., Lim B.-S., Kim C.-W. (2002). Effect of surface conditions on the color of dental resin composites. *Journal of Biomedical Materials Research*.

[11] Bağış B., Bağış H. Y. (2006). Porselen laminate veneerlerin klinik uygulama aşamaları: klinik bir olgu sunumu. *Ankara Üniversitesi Diş Hekimliği Fakültesi Dergisi*.

[12] Hickel R., Heidemann D., Staehle H. J., Minnig P., Wilson N. H. (2004). Direct composite restorations: extended use in anterior and posterior situations. *Clinical Oral Investigations*.

[13] Khashayar G., Dozic A., Kleverlaan C. J., Feilzer A. J., Roeters J. (2014). The influence of varying layer thicknesses on the color predictability of two different composite layering concepts. *Dental Materials*.

[14] Magne P., Belser U. C. (2003). Porcelain versus composite inlays/onlays: effects of mechanical loads on stress distribution, adhesion, and crown flexure. *The International Journal of Periodontics & Restorative Dentistry*.

[15] Berksun S., Kedici P. S., Saglam S. (1993). Repair of fractured porcelain restorations with composite bonded porcelain laminate contours. *The Journal of Prosthetic Dentistry*.

[16] Jordan R. E. (1993). *Esthetic Composite Bonding Techniques and Materials*.

[17] Stappert C. F. J., Ozden U., Gerds T., Strub J. R. (2005). Longevity and failure load of ceramic veneers with different preparation designs after exposure to masticatory simulation. *Journal of Prosthetic Dentistry*.

[18] Hemmings K. W., Darbar U. R., Vaughan S. (2000). Tooth wear treated with direct composite restorations at an increased vertical dimension: results at 30 months. *The Journal of Prosthetic Dentistry*.

[19] Garber D. A., Goldstein R. E., Feinman R. A. (1988). *Porcelain Laminate Veneers*.

[20] Bağış B., Bağış H. Y. (2006). Porselen laminate veneerlerin klinik Uygulama aşamaları: Klinik bir olgu sunumu. *Ankara Üniversitesi Diş Hekimliği Fakültesi Dergisi*.

[21] Azzaldeen A., Muhamad A. H. (2015). Diastema closure with direct composite: architectural gingival contouring. *Journal of Advanced Medical and Dental Sciences Research*.

[22] Demirci M., Tuncer S., Öztaş E., Tekçe N., Uysal Ö. (2015). A 4-year clinical evaluation of direct composite build-ups for space closure after orthodontic treatment. *Clinical Oral Investigations*.

